# Field testing of a multicriteria decision analysis (MCDA) framework for coverage of a screening test for cervical cancer in South Africa

**DOI:** 10.1186/1478-7547-10-2

**Published:** 2012-02-29

**Authors:** Jacqui Miot, Monika Wagner, Hanane Khoury, Donna Rindress, Mireille M Goetghebeur

**Affiliations:** 1 Division Clinical Epidemiology, School of Health Systems and Public Health, Faculty of Health Sciences, University of Pretoria, Pretoria, South Africa; 2 BioMedCom Consultants inc, Montreal, QC, Canada

## Abstract

**Background:**

Systematic and transparent approaches to priority setting are needed, particularly in low-resource settings, to produce decisions that are sound and acceptable to stakeholders. The EVIDEM framework brings together Health Technology Assessment (HTA) and multi-criteria decision analysis (MCDA) by proposing a comprehensive set of decision criteria together with standardized processes to support decisionmaking. The objective of the study was to field test the framework for decisionmaking on a screening test by a private health plan in South Africa.

**Methods:**

Liquid-based cytology (LBC) for cervical cancer screening was selected by the health plan for this field test. An HTA report structured by decision criterion (14 criteria organized in the MCDA matrix and 4 contextual criteria) was produced based on a literature review and input from the health plan. During workshop sessions, committee members 1) weighted each MCDA decision criterion to express their individual perspectives, and 2) to appraise LBC, assigned scores to each MCDA criterion on the basis of the by-criterion HTA report.

Committee members then considered the potential impacts of four contextual criteria on the use of LBC in the context of their health plan. Feedback on the framework and process was collected through discussion and from a questionnaire.

**Results:**

For 9 of the MCDA matrix decision criteria, 89% or more of committee members thought they should always be considered in decisionmaking. Greatest weights were given to the criteria "Budget impact", "Cost-effectiveness" and "Completeness and consistency of reporting evidence". When appraising LBC for cervical cancer screening, the committee assigned the highest scores to "Relevance and validity of evidence" and "Disease severity". Combination of weights and scores yielded a mean MCDA value estimate of 46% (SD 7%) of the potential maximum value. Overall, the committee felt the framework brought greater clarity to the decisionmaking process and was easily adaptable to different types of health interventions.

**Conclusions:**

The EVIDEM framework was easily adapted to evaluating a screening technology in South Africa, thereby broadening its applicability in healthcare decision making.

## Background

In any country, healthcare resource allocation decisions are complex and involve assessment of the available scientific evidence, clarification of priorities, value judgments and ethical considerations [1,2]. In developing countries, which are generally low-resource settings, priority setting for healthcare becomes even more important. Not only are resources limited, but there are also other factors such as poor information, lack of policy, barriers to implementation, and political agendas, to name but a few [3]. The result is that priority setting is inconsistent and unstructured [1]. Transparent and explicit approaches to decisionmaking help produce decisions that are sound and acceptable to stakeholders [4-7].

In the healthcare sector of South Africa, current decisionmaking approaches are centered around evidence-based medicine, affordability and, where available, cost-effectiveness/costutility analysis (CEA/CUA) [8]. Increasingly CEA/CUA is being used for priority setting at all levels: the patient; the healthcare service; and within populations. This is evident in the field of HIV/AIDS where cost-effectiveness analyses of antiretroviral (ARV) medicines has enhanced access to treatment and reduced drug prices [9,10]. In many instances, a simple cost-minimization approach is all that is attempted. However, this approach has shortcomings, as there are a number of additional important dimensions, such as budget impact, equity, availability of alternatives, disease severity, etc. [11,12], that are not incorporated. Where these are taken into consideration, they are often assessed in an ad hoc manner and there is a lack of transparency as to how they impact the final decision [1].

Thus, there is a need for a process that supports consideration of all dimensions impacting a decision in a systematic and explicit fashion, and increases transparency and access to the evidence upon which decisions are based. Multi-criteria decision analysis (MCDA) is a tool to support complex decisionmaking which allows a structured, objective consideration of factors that are both measurable and value-based in an open and transparent manner [1,13,14].

While MCDA has been used historically in sectors such as transport or agriculture, there is a growing interest in using and applying the principles of MCDA, and similar approaches based on multiple decision criteria, to resource allocation decisionmaking in health care [15-21]. The EVIDEM framework has been developed to bring together Health Technology Assessment (HTA) and MCDA by proposing a comprehensive set of decision criteria together with standardized processes/methods to develop HTA reports that are structured on these criteria [22]. The aims of the framework are to facilitate concurrent consideration of multiple decision criteria, to stimulate reflection on priorities and values, and to promote transparency and communication within the decisionmaking committee as well as with outside stakeholders. The application of the EVIDEM framework is postulated to be wide ranging from decisionmaking by the healthcare provider, to coverage decisions by the funders or government policy setting.

A proof-of-concept study of the EVIDEM framework was performed in Canada involving a diverse panel of stakeholders appraising 10 medicines [23]. The core framework was also further developed to include standardized contextual criteria and tested for clinical decisionmaking by a panel of pediatric endocrinologists and other stakeholders who applied it to appraise growth hormone for children with Turner syndrome in Canada [24]. The framework was also tested as a support for drug formulary decisionmaking by a public healthcare payer in Canada [25].

The objective of the current study was to expand the scope of field testing both geographically and with respect to type of intervention: i.e., to field test the framework as a support for coverage decisionmaking on a cervical cancer screening test (liquid-based cytology, LBC) by a private health plan in South Africa.

The healthcare system in South Africa is dichotomous with a small (approximately 7 million lives) but resource-rich private healthcare sector and a growing (approximately 45 million lives) resource-scarce public sector. The private healthcare sector is largely funded by medical insurance companies (health plans), while the public sector is currently funded out-of-pocket or by the government. With respect to cervical cancer screening, the Department of Health guidelines stipulate that all women should be screened three times in their lifetime from the age of 30 years [26]. In the private healthcare sector, cervical cancer screening is often recommended and carried out on an annual basis. Screening uptake is low in South Africa with poor accessibility to healthcare facilities, poorly trained staff, long turnaround times between laboratory and healthcare facility, as well as lack of education cited as some of the reasons [27]. LBC for cervical cancer screening was selected by the health plan as a relevant case study due to its recent introduction as an alternative to conventional Pap smears.

## Methods

### Study design

The EVIDEM framework was field-tested in the private healthcare sector of South Africa with a clinical policy and decisionmaking committee of a major health plan. This panel of experts included doctors (specialists and general practitioners), pharmacists and nurses with at least 12 months experience in decision-making at a health policy level for the health plan. At the time of the study, the committee was already using evidence-based medicine, cost-effectiveness, affordability and some epidemiological principles in a multicriteria-based decisionmaking process.

**Figure 1:**
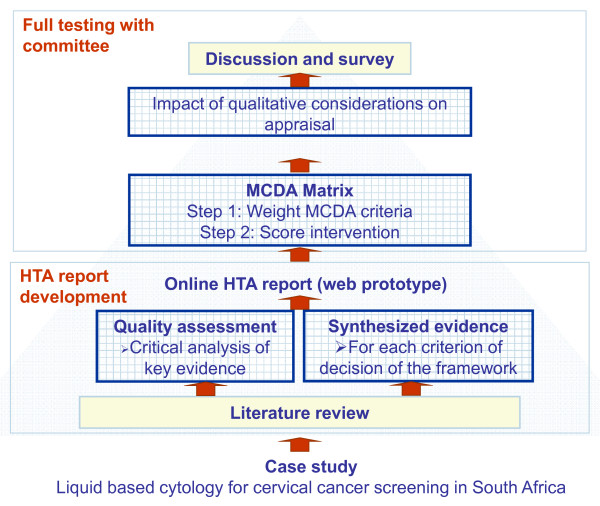
**Study plan**.

The study design is presented in Figure 1. Based on a literature review and input from the clinical committee of the health plan, a structured HTA report on LBC for cervical cancer screening was produced and tailored to investigate each of the 14 MCDA decision criteria of the framework organized in the MCDA matrix [22]. (Note that the framework contains 15 MCDA decision criteria; however, the criterion "Adherence to requirements of decisionmaking body" was not considered for appraisal in this field test.) Four contextual criteria were proposed by the healthcare funder: "Impact on future decisions", "Relationship with pathology providers", "Impact on screening intervals" and "Patient expectation"; these criteria were appraised qualitatively. The contents of the report were tailored to reflect the local context (i.e., South African private health plan). Each committee member was familiarised with the LBC technology through prior evaluation for clinical decisionmaking and/or review of the HTA report.

During workshop sessions, committee members assigned: 1) weights to each criterion of the MCDA matrix to express their perspectives; 2) scores for LBC for each criterion of the MCDA matrix based on data from the by-criterion HTA report; and 3) the qualitative impact of system-related criteria on the appraisal. The adoptability and utility of the EVIDEM approach were explored through a post-testing survey.

### Health technology assessment report

Relevant data for each decision criterion of the framework was identified by searching PubMed, websites of HTA agencies (including the Canadian Agency for Drugs and Technologies in Health [CADTH], National Institute for Health and Clinical Excellence [NICE], US Preventative Services Task Force and Danish Centre for Evaluation and Health Technology Assessment) and the website of South Africa's Department of Health.

Data on the projected budget impact of LBC as well as local costing data was obtained from the health plan. The health plan also provided input for the four contextual criteria, which were appraised qualitatively. Collected data was extracted, analyzed and synthesized to produce a by-criterion HTA report.

The quality of the clinical evidence and the economic evaluation was assessed using previously developed instruments according to two quality criteria: "Completeness and consistency or reporting" and "Relevance and validity of evidence" [22]. The HTA report was entered into an interactive web prototype (Tikiwiki v2.2) that was hyperlinked to the full-text sources from which data was extracted. The web prototype was designed to allow online appraisal of the proposed intervention and collect feedback from the committee.

### Field testing by the committee

To explore their individual values, committee members were asked to assign weights to each MCDA decision criterion on a scale of 1 to 5, where 5 represents the greatest importance to their decisionmaking. Weights were to be assigned from individual perspectives in the context of the health plan, independent of the intervention to be appraised.

All committee members were asked to familiarise themselves with the study intervention (e.g. clinical trials, health economic studies, disease characteristics and treatments etc.) using the EVIDEM Synthesised evidence report prior to carrying out the evaluation.

To appraise the proposed intervention, committee members were then asked to score (on a 4-point scale from 0 to 3) LBC for cervical cancer screening with respect to each MCDA criterion of decision, based on synthesized data from the by-criterion HTA report. Committee members then considered what type of impact each of the four qualitative criteria might have on use of LBC in the context of the health plan.

Feedback on the framework and process was elicited during discussion periods at the first workshop and at a follow-up workshop, and from a questionnaire administered during the follow-up workshop. Committee members were surveyed about whether each of the framework's decision criteria should always, sometimes or never be considered in decisionmaking. They were also surveyed about how the EVIDEM approach compared to their current process regarding data provided, deliberative process and communication of decision (improved, same or worse).

### Data collection and analyses

Weights, scores and impact obtained from committee members were entered into Excel software. Descriptive statistics were used and mean ± standard deviations (SD) were calculated for weights and scores. The MCDA value estimate for the use of LBC in cervical cancer screening was then obtained by applying an MCDA linear additive model [22]. Thus, the value contribution of each decision criterion was calculated by multiplying its mean weight (normalized across the criteria to add up to 1 for each committee member) and score (standardized by division by the maximum score 3). The MCDA value estimate was then calculated as the sum of the value contributions of the 14 applicable criteria of the MCDA matrix.

## Results

### Decision criteria and committee values

When surveyed on whether each of the criteria of the framework should always, sometimes or never be considered in the decisionmaking process, all committee members agreed that "Budget impact on health plan" should *always *be considered (Table 1). For eight further criteria ("Completeness and consistency of reporting evidence", "Relevance and validity of evidence", "Disease severity", "Clinical guidelines", "Improvement of efficacy/effectiveness", "Type of medical service", "Cost-effectiveness" and "Appropriate use"), eight out of nine (89%) committee members felt that these should *always *be considered. Among the 15 MCDA matrix criteria, "Improvement of PRO, convenience & adherence" and "Adherence to requirements of decisionmaking body" received the lowest acceptance from committee members, with only 44% and 33%, respectively, indicating that these should *always *be considered. "Political context" received least approval among all criteria included in the survey, with only one (11%) committee member expressing the opinion that it should *always *be considered. No committee member indicated that any criterion should *never *be considered.

**Table 1:** Committee member responses to whether specific decision criteria should be considered in decision making

Criteria	Total number of responses	Distribution of responses		
		**Always**	**Sometimes Never**	

**MCDA matrix criteria**				

**Quality of evidence**				

Adherence to requirements of decision making body	9	33	67	0

Completeness and consistency of reporting evidence	9	89	11	0

Relevance and validity of evidence	9	89	11	0

**Disease impact**				

Disease severity	9	89	11	0

Size of population affected by disease	9	67	33	0

**Intervention**				

Current clinical guidelines	9	89	11	0

Current interventions limitations	9	78	22	0

Improvement of efficacy/effectiveness	9	89	11	0

Improvement of safety & tolerability	9	78	22	0

Improvement of PRO, convenience & adherence	9	44	56	0

Public health interest (prevention & risk reduction)	9	78	22	0

Type of medical service	9	89	11	0

**Economics**				

Budget impact on health plan	9	100	0	0

Cost-effectiveness of intervention	9	89	11	0

Impact on other spending	9	78	22	0

**Other criteria**				

Appropriate use	9	89	11	0

Opportunity costs	9	78	22	0

Organizational structure	9	22	78	0

Stakeholder pressures	9	33	67	0

Political context	9	11	89	0

Population priorities and access	9	22	78	0

Regulatory status of intervention	9	56	44	0

**Figure 2:**
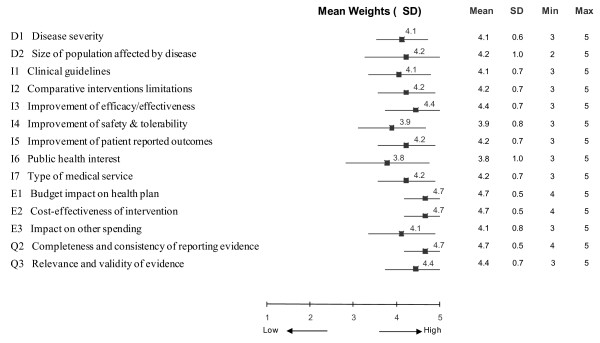
**Mean weights assigned to each decision criterion of the MCDA matrix by committee members**. A five point weighting scale was used with 1 for lowest weight and 5 for highest weight.

Committee members were asked to weight each of the 14 MCDA decision criteria included in this field test according to their importance to the appraisal of an intervention, independently of the intervention that was to be appraised. (The criterion "Adherence to requirements of decisionmaking body" was not considered for appraisal in this field test.) The greatest weights (mean 4.7, on a scale of 1 to 5) were assigned to the criteria "Budget impact on health plan", "Cost-effectiveness of intervention" and "Completeness and consistency of reporting evidence", followed by "Improvement of efficacy/effectiveness" and "Relevance and validity of evidence" (mean 4.4) (Figure 2). The lowest weights were given to "Improvement of safety and tolerability" (mean 3.9) and "Public health interest" (mean 3.8). Weights for "Budget impact on health plan", "Cost-effectiveness of intervention" and "Completeness and consistency of reporting evidence" showed the least variation among committee members (SD 0.5). The largest divergence of weights was recorded for the criteria "Size of population affected" (SD 1.0) and "Public health interest" (SD 1.0).

### Appraisal of liquid based cytology for cervical cancer screening

#### By-criterion Health Technology Assessment report

The HTA report for LBC was based on 14 references (Table 2, 'lite' version). (The full report is available online at http://www.evidem.org/tiki/?page=CCLBC-RecordMenu). Key items included in the assessment are reported here. In the context of cervical cancer screening, LBC refers to a technique in which the cervical sample is suspended into a preservative liquid, rather than, as in conventional cytology (CC, Pap test), being smeared onto a slide. Cervical cancer causes high morbidity and mortality [28-30]. With annual incidence rates of 9 per 100,000 among white and 40 per 100,000 among black women [31], cervical cancer is the second most common cause of cancer morbidity and mortality in South African women [32-34]. Current South African guidelines recommend screening with at least three cervical Pap smears (CC) per lifetime to detect precancerous lesions; they do not mention LBC [26]. In addition to the occurrence of unsatisfactory samples, the chief limitations of CC are its relatively low specificity and sensitivity and its low uptake by the population [27,35,36]. There is considerable public health interest in improving screening methods and uptake to reduce the high burden of cervical cancer in South Africa [32,33]. As a screening tool, LBC allows early detection which, with appropriate treatment, improves outcomes [30]. A meta-analysis of RCTs found that LBC had 6.4% higher sensitivity but 4.0% lower specificity than CC, coupled with a slight reduction in the percentage of unsatisfactory samples [35]. The most relevant outcomes, impact on morbidity and mortality due to cervical cancer, have not been measured. The incremental budget impact of LBC is estimated to be ZAR76.90 per patients screened and the incremental colposcopy cost per patient lifetime ZAR8 [36,37]. The incremental cost per QALY gained was projected to be ZAR758,000 with a cost of ZAR764,000 per life-year gained [35]. However, since these data are based on a Canadian economic evaluation model they may have limited relevance to the South African setting.

**Table 2:** HTA report with data for each decision criterion of the EVIDEM framework ('Lite' highly synthesized version)*

Overview						
**Disease:**	**Type of intervention**: Cervical cytology test					
Liquid Based Cytology (LBC)						

**Intervention:**	**Indication**: Screening for cervical cancer in women					
Cervical cancer						

**Setting:**	**Administration**: Liquid based cytology (LBC) requires physician to obtain					

Discovery Health, South Africa	cells from the cervix and to place the head of the spatula/brush or rinse it into a vial with preservative fluid. The pathologist then extracts the cells into a microscopy slide and the cells are examined in the usual way					

	**Comparator(s)**: Conventional pap smears (i.e., conventional cytology, CC) where the brush containing the sample is smeared onto the slide, thereby transferring the cells					

	**Data included:**Data available from public domain					

**MCDA matrix criteria**	**Highly synthesized information**		**Scoring of intervention**			

**Disease impact**			Not severe		Very severe	

D1	Disease severity	**Disease symptomatic only when spreading from cervix: **vaginal bleeding, post-coital spotting, vaginal discharge, pelvic or low back pain.	0	1	2	3
						
		**Late stages**: severe anemia, weight loss and uncontrolled release of urine and feces through the vagina				
						
		**Survival**: < 18 months in 50% of untreated patients				

D2	Size of population	**Incidence in South Africa**: 9/100,000 white women; 40/100,000 black women	Very rare disease		Very common disease	
			
			0	1	2	3

**Intervention**						

I1	Clinical guidelines	LBC is not included in any guidelines.**South African guidelines **recommend at least 3 pap smears test per lifetime	Not recommended		Strong recommendation	
			
			0	1	2	3

I2	Comparative interventions limitations	**Sensitivity**: **74% **- relatively low; **Specificity**: **87%**;**Unsatisfactory sample**: **3%Low screening uptake: **2.8% black women; 18.8% white women	No or very minor limitations		Major limitations	
			
			0	1	2	3

I3	Improvement of efficacy/effectiveness	**Meta-analyses of RCTs **(n = 28,736 vs 39,377): **Sensitivity: **80% - difference with CC = 6.4% (95% CI: -6.5 to 18.8%)**Specificity: **82% - difference with CC = - 4.0% (95% CI: -19.9 to 10.6%) **Unsatisfactory samples**: **ThinPrep = 2.2% **and **Sure Path = 0.82%**; difference with CC: ThinPrep = -0.8% and Sure Path = -2.5%	Lower than comparators		Major improvement	
			
			0	1	2	3

I4	Improvement of safety & tolerability	**Safety and tolerability **do not differ between LBC and conventional cytology	Lower than comparators		Major improvement	
			
			0	1	2	3

I5	Improvement of patient reported outcomes	**Patient reported outcomes**: No data - fewer recalls and fewer inadequate specimens with LBC may improve quality of life**Convenience**: no need to return for HPV testing in case of a positive result	Lower than comparators		Major improvement	
			
			0	1	2	3

I6	Public health interest	**Screening programs for cervical cancer in South Africa**: mortality decreased by 50% for white women, 40% for Asian women but rose for black women between 1960's 1990's. and Considerable interest to improve screening methods and uptake.	No risk reduction		Major risk reduction	
			
			0	1	2	3

I7	Type of medical service	**Goal of intervention**: improve outcome of the disease due to early detection; 5-years survival ranging between close to 100% for early stage and 5% to 15% for late stage	Minor Service		Major Service *(e.g. cure)*	

**Economics**						

E1	Budget impact on health plan	**Intervention price: **LBC - R180.00; CC - R103.10**Incremental cost per pt screened with LBC**: R76.90**Annual projected budget impact per 1000 women**: R76,900	Substantial additional spending		Substantial savings	
			
			0	1	2	3

E2	Cost-effectiveness of intervention	**Cost per life-year gained**: R764,000**Cost per QALY gained**: R758,000	Not cost-effective		Highly cost-effective	
			
			0	1	2	3

E3	Impact on other spending	**Incremental colposcopy cost per patient over lifetime horizon**: R 8 (excludes drug cost, see E1)No available data on **other spending **(including treatment of cancer and pre-cancer)	Substantial additional spending		Substantial Savings	
			
			0	1	2	3

**Quality of evidence**						

*Q1*	*Adherence to requirements of decisionmaking body*	*Not applicable for case study*	Low adherence		High adherence	

Q2	Completeness and consistency of reporting evidence	**Quality score: Clinical data:75% **- primary and secondary outcome measures as well as sensitivity analyses not clearly specified; **Economic evaluation: 50% **- disaggregated cost not reported; incomplete reporting of effectiveness outcomes	Many gaps/inconsistent		Complete & consistent	
			
			0	1	2	3

Q3	Relevance and validity of evidence	**Quality score: Clinical data:75% **-most relevant outcome not assessed (morbidity and mortality due to cervical cancer);**Economic evaluation: 75% **- Canadian screening coverage and HPV epidemiology not completely applicable for South African private payer setting	Low relevance/validity		High relevance/validity	
			
			0	1	2	3

* The full HTA report is available online at http://www.evidem.org/tiki/?page=CCLBC-RecordMenu

#### Quantitative considerations - scores

**Figure 3:**
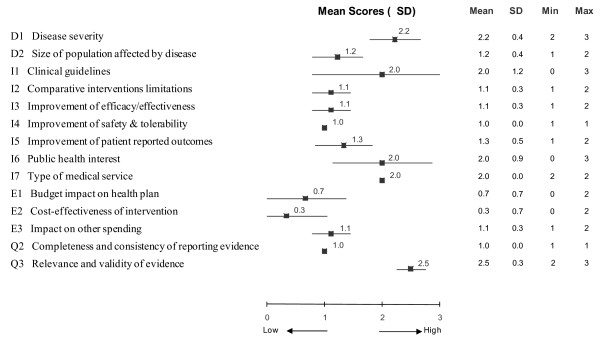
**Mean scores assigned to each decision criterion of the MCDA matrix by committee members for the appraisal of liquid based cytology**. A four point scoring scale was used with 0 for lowest score and 3 for highest score.

Using synthesized data from the HTA report in the MCDA matrix (Table 1), committee members assigned scores to appraise LBC for each criterion (Figure 3). The highest scores were assigned to the criteria "Relevance and validity of evidence" (mean 2.5, SD 0.3, on a scale of 0 to 3) and "Disease severity" (mean 2.2, SD 0.4). The lowest scores were given to "Cost-effectiveness of intervention" (mean 0.3, SD 0.7), "Improvement of safety & tolerability" (mean 1.0, SD 0.0) and "Completeness and consistency of reporting evidence" (mean 1.0, SD 0.0). The committee expressed unanimous agreement (SD 0.0) when scoring the criteria "Improvement of safety & tolerability" (mean 1.0), "Completeness and consistency of reporting evidence" (mean 1.0), and "Type of medical service" (mean 2.0). In contrast, 3-point differences across the full scoring scale (0 to 3) were seen for the criteria "Clinical guidelines" (mean 2.0, SD 1.2) and "Public health interest" (mean 2.0, SD 0.9).

#### MCDA value estimate

**Figure 4:**
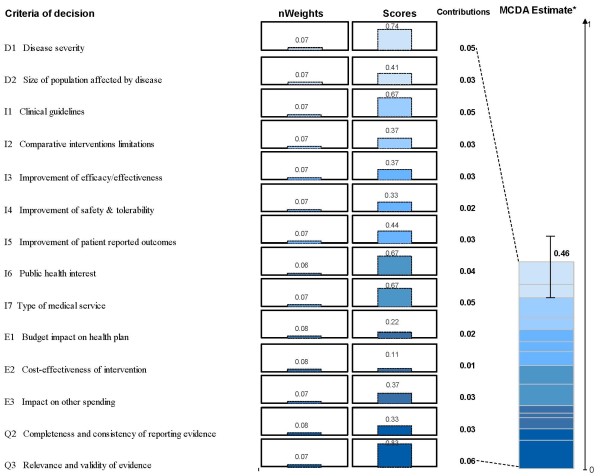
**MCDA value estimate of liquid based cytology for cervical cancer screening based on weights and scores assigned by committee members**. Weights were normalized across the 14 criteria and scores are presented on a scale of 0 to 1. *MCDA estimate was obtained using a linear model combining normalized weights and scores for each decision criteria. For an intervention to achieve close to 1 on this scale, it would have to cure an endemic disease, demonstrate major improvement in safety, efficacy, and patient reported outcomes compared to limited existing approaches and result in major healthcare savings.

Combination of the committee's weights and scores yielded a mean MCDA value estimate for using LBC in cervical cancer screening of 46% (SD 7%) of the potential maximum value (Figure 4). Individual MCDA value estimates ranged from 36% to 61% among the nine committee members; 7 out of 9 estimates were in the range between 39% and 48%. The criterion "Relevance and validity of evidence" made the greatest contribution to the mean MCDA value estimate (13% of the total), followed by "Disease severity", "Clinical guidelines" and "Type of medical service" (11% each). The criterion "Cost-effectiveness" made the smallest contribution (2%) to the MCDA value estimate.

#### Qualitative considerations - impacts

Background information for the four contextual criteria proposed by the health plan was collected in the context of the health plan and provided to the committee to stimulate reflection and discussion (Table 3). Committee members considered that a decision to cover LBC may have a negative impact on future decisions (setting a precedent). When considering relationships with pathology providers, covering LBC was considered to have a positive impact. The possibility of extending screening intervals would also have a positive impact on the value of LBC. When considering patient expectations, committee members considered that patients would likely resist if co-payments were implemented for LBC.

**Table 3:** Qualitative considerations and their impact on the appraisal of LBC by committee members

Contextual criteria*	Should this be considered? Would it impact positively or negatively on value of intervention?
Impact on future decisions	Decision to fund LBC coverage although not considered cost-effective will be setting a precedent as LBC technology will be expanded to other cytology tests Considered as negative impact on value

Relationship with pathology providers	Ongoing negotiations with the pathology groups in other areas may be impacted Considered as a positive impact on value

Impact on screening intervals	Screening interval may be extended to 2 to 3 years than annually Considered as a positive impact on value

Patient expectation	Health plan members expect that their pap smears will be paid in full and are likely to resist any benefit design which implements a co-payment for a more expensive technology Considered as a negative impact on value

*Contextual criteria were proposed by the healthcare funder. Specific contextual criteria may be defined on the basis of the following generic criteria/themes: mission and scope of the healthcare system, opportunity costs; population priorities & access; system capacity and appropriate use; political context; and stakeholder pressures.

### Feedback from committee on overall approach

A feedback questionnaire was completed by the committee members following the evaluation. The elements of the questionnaire were pre-determined and part of the standardised set of tools available for the EVIDEM process. When surveyed on how the EVIDEM approach compared to existing process that committee members were familiar with, 50% of the committee members felt that it improved understanding of the intervention being appraised, access to quality assessment of the evidence on the intervention, and consideration of all key elements of the decision (Table 4). Fifty-six percent of committee members felt that the framework improved the transparency of the decision and the communication of the decision to stakeholders. No committee member rated the EVIDEM process as "worse". Concern was raised regarding the time and resources required to generate the HTA report, but on the whole, the committee felt it was a positive step forward in improving decisionmaking by bringing greater clarity to the decisionmaking process. The committee also thought that the approach could easily be applied to evaluation of other health interventions such as medicines and devices.

**Table 4:** Feedback from committee members on the EVIDEM process compared to current approaches

Component	Total number of responses	Distribution responses (%)		
		**Improved**	**Same**	**Worse**

**Intervention under scrutiny**				

Understanding of intervention	8	50	50	0

Access to evidence on intervention	8	25	75	0

Access to quality assessment of evidence on intervention	8	50	50	0

**Deliberative process**				

Considering all key elements of decision	8	50	50	0

Expressing personal/expert opinion	9	11	89	0

Sharing & discussing values among committee members	9	22	78	0

**Communication of decision**				

Transparency of decision	9	56	44	0

Understandability of decision by stakeholders	9	56	44	0

Acceptability of decision by stakeholders	7	43	57	0

The EVIDEM process for LBC resulted in a consideration by the health plan to only fund for LBC up to the value of conventional pap smears. A negotiation process was started with the pathology laboratories to review their tariffs for this diagnostic. The fee for LBC was reduced to an amount which was considered appropriate for full funding.

## Discussion

The EVIDEM framework can be applied to assess the value of a screening intervention in the South African private health plan context, providing a practical tool for integrating HTA with an MCDA approach to support decisionmaking.

The use of the by-criterion HTA report allowed the clinical decisionmaking panel to assess all the criteria easily and from a single source. Where greater detail was required, hyperlinks embedded in the web-prototype provided access to the more detailed synthesized information as well as full source data. Available clinical evidence was fairly relevant and valid. Partial reporting of disaggregated data in the published economic evaluation limited its usefulness in the South African context although local costs were used to improve transferability. The fact that the committee gave the highest weighting to "Budget impact on health plan", "Cost-effectiveness of intervention" and "Completeness and consistency of reporting evidence" reflects its focus on issues of affordability and cost-effectiveness as well as quality of clinical evidence. This was also reflected by the low score (mean 0.3) the committee assigned to the cost-effectiveness of LBC (Figure 3), given an ICER of ZAR764,000 per QALY.

The MCDA estimate resulting from a combination of weights and scores, provided a comprehensive measure of the perceived value of LBC in the current context, i.e., relative to existing technology (conventional cytology), reflecting minor improvement at a significant cost, and also captured the importance of absolute elements of value such as disease severity, type of medical service and quality of evidence.

Non-quantifiable, contextual criteria of decision were also identified and considered. Given the lack of cost-effectiveness and high additional cost of LBC compared to conventional Pap smears, the budget impact would be substantial and therefore a co-payment would be considered. However, health plan members and many other South Africans are used to receiving Pap smears for free as part of health screening programmes and the implementation of co-payments would meet with strong resistance from both patients and healthcare providers. The use of the MCDA framework as a communication device to convey the structure and transparency of the decisionmaking process was thought to be an important tool to improve acceptability of the committee's decision.

Adaptation of the EVIDEM framework from medicines evaluation to a screening technology required minimal change to the structure of the framework. Since devices, procedures and technologies form a growing portion of the basket of healthcare interventions that require evaluation it was useful to assess the adaptability of the framework to these types of interventions.

Currently, there is no single HTA body (such as NICE or CADTH) in South Africa with the result that evaluations of new and existing health interventions are scattered and often duplicated. Skilled resources to carry out a proper HTA are scarce and a need is recognized for pooling these resources, allowing greater availability to all sectors in the country [38,39]. Health technology assessments and reports are freely available from a number of sources such as NICE (UK), CADTH (Canada), AHRQ (USA), IQWIG (Germany), etc., as well as through subscription to knowledge bases such as the Cochrane Collaboration. However, none of these reports offers the opportunity to rationally and transparently measure the impact of the various criteria on the final clinical decision. This is left to clinical decisionmaking members who are intrinsically subjective based on their own priorities and knowledge. Using MCDA in healthcare decisionmaking adopts an existing, well established methodology based on sound, scientific principles [40].

On the whole, the committee members favoured the use of the EVIDEM process and were positive about its use. Some committee members rated their feedback of the EVIDEM process as "same" as existing process in the questionnaire, which may be explained by the fact that a more crude and simpler multicriteria approach was already being used by the committee.

This field test indicates that the EVIDEM framework may be useful for the evaluation of health technologies in the South African private healthcare sector context. However, further adaptation to a health plan's specific mission, scope and priorities is required to create a "custom-made" decisionmaking framework fully aligned with the local context. The framework's contextual tool ("extrinsic value tool") -- a result of a further development of this framework [24] -- facilitates this customization by providing generic criteria/themes on the basis of which contextual criteria can be identified, and if so desired, integrated into the MCDA model. Further development and validation of the framework's weighting and scoring methodologies is also currently underway.

## Conclusions

The EVIDEM framework was easily adapted to evaluating a screening technology thereby broadening its applicability in healthcare evaluation. It was found to improve: understanding of intervention; access to quality assessment of evidence; consideration of key elements of decision; transparency of decision; and understandability of decision by stakeholders. Further field testing and instrument validation and development are ongoing to collaboratively advance MCDA approaches and contribute to more transparent and efficient healthcare decisionmaking.

## Abbreviations

AEs: Adverse events; ANOVA: Analysis of variance; CE: Cost-effectiveness; EVIDEM: Evidence and Value: Impact on DEcisionMaking; HTA: Health technology assessment; ICC: Intra-rater correlation coefficients; INAHTA: International Network of Agencies for Heath Technology Assessment; MCDA: Multicriteria decision analysis; PRO: Patient reported outcomes; QALY: Quality-adjusted life-year; QM: Quality Matrix; RCT: Randomized controlled trial; SD: Standard deviation.

## Competing interests

The authors declare that they have no competing interests.

## Authors' contributions

JKM, MMG, and MW designed the study and HK collected and analysed the data. JKM, MMG and MW drafted the manuscript. DR reviewed the manuscript and is one of the developers of the EVIDEM framework. All authors read and approved the final manuscript.
